# Traditional Chinese medicine for Helicobacter pylori infection: A protocol for a systematic review and meta-analysis

**DOI:** 10.1097/MD.0000000000024282

**Published:** 2021-01-22

**Authors:** Mao Zhao, Yuchang Jiang, Zhaoxing Chen, Zhipeng Fan, Yong Jiang

**Affiliations:** Chengdu University of Traditional Chinese Medicine, School of Basic Medical Sciences, Chengdu, China.

**Keywords:** Helicobacter pylori, protocol, systematic review and meta-analysis, Traditional Chinese medicine

## Abstract

**Background:**

Helicobacter pylori (Hp) is the only bacterium in the stomach. It is characterized by its ability to adhere to gastric mucosa and cause a series of pathological changes in the gastric mucosa. Modern research shows that Hp is an important pathogenic factor for chronic gastritis, gastroduodenal ulcer, and gastric cancer. Triple, quadruple, and triple combinations of antibacterial drugs, proton pump inhibitors, and bismuth aluminate preparations have been developed in modern medical research. Sequential therapy is used to treat Hp, but antibiotic resistance and repeated infections still exist. A large number of clinical trials have proved that traditional Chinese medicine has a good therapeutic effect on Hp. In this systematic review, we aim to evaluate the efficacy and safety of traditional Chinese medicine in the treatment of Hp.

**Methods and analysis:**

We will search for publications from Web of Science, PubMed, Science Direct, Wan Fang Data Knowledge Service Platform, Chinese Biomedical Literature Database (CBM), Chinese Scientific Journal Database (VIP database), China National Knowledge Infrastructure (CNKI) and EMBASE, which should be published from inception to December 2020. Two researchers will independently perform the selection of the studies, data extraction, and synthesis. The Cochrane Risk of Bias Tool will be used to evaluate the risk of bias in the randomized controlled trials. Statistical analysis will be performed by using the Cochrane Review Manager (RevMan 5.3) software. The *I*^2^ test will be used to identify the extent of heterogeneity. We will use the Egger funnel chart to evaluate possible publication biases, in addition, when possible we will perform a subgroup/meta-regression analysis. The strength of the evidence will be assessed according to the Grading of Recommendations Assessment, Development, and Evaluation (GRADE).

**Results and conclusions:**

This study will systematically evaluate the efficacy of traditional Chinese medicine in the treatment of Hp infection, and provide evidence for the clinical application of this treatment. The results of the research will be published in a peer-reviewed journal.

**Ethics:**

This systematic review will evaluate the efficacy of traditional Chinese medicine for Hp infection. Because all data used in this systematic review and meta-analysis have been published, this review does not require ethical approval.

**Trial registration number:**

INPLASY2020120057.

## 1 Introduction

In 1983, Australian scientists Warren and Marshall found and isolated Helicobacter pylori (Hp) in human gastric mucosa.^[1,2]^ Public health studies have shown that Hp, as a pathogen, only spreads from person to person. In addition to mouth-to-mouth, fecal-oral and iatrogenic routes, Hp can also be spread through water. Because of its wide transmission route, it is easy to get sick, highly contagious and spread widely.^[1,3]^ The incidence rate in developing countries is higher than that in developed countries, The global population infection rate is as high as 50%, which has been on the rise in recent years.^[4–6]^ It has been found in the laboratory that Hp is a Gram-negative bacterium, which can cause damage and degradation of gastric mucosa by adhering to gastric mucosa and urease and its release of cytotoxin, protease and phospholipase.^[7–9]^ Etiology and pathology studies have proved that Hp is an important pathogenic factor of chronic gastritis, gastroduodenal ulcer, intestinal metaplasia, and gastric cancer.^[10–14]^ Therefore, as early as 1994, the World Health Organization listed this bacteria as a Class I carcinogen.^[15]^ Hp infection develops into chronic gastritis at an early stage and may develop into precancerous lesions and intestinal metaplasia of atrophic gastritis, among which gastric cancer is the most serious result.^[9]^ Gastric cancer is a major global health threat and it is also the third leading cause of cancer deaths worldwide. It is estimated that more than 720,000 people die each year worldwide.^[6]^

Therefore, Hp infection has great influence on society and economy, and eradication of Hp has become the main goal of digestive system diseases. Eradication of Hp can promote the alleviation of gastric inflammation, advance the damage of gastric mucosa, prevent further DNA damage caused by Hp, improve gastric acid secretion and restore the normal microbial population. For Hp infection, modern medicine adopts triple, quadruple and sequential treatment based on antibacterial drugs, proton pump inhibitors and bismuth aluminate preparations. The treatment effect is to direct sterilization, protection of gastric mucosa and neutralization of gastric acid.^[16]^ However, with the abuse of antibiotics, pure western medicine treatment is faced with many problems, such as high drug resistance, great toxic and side effects, intestinal flora imbalance, high recurrence rate, etc.^[3,17–22]^

With the wide application of traditional Chinese medicine in the treatment of Hp, a large number of clinical and pharmacological studies of traditional Chinese medicine show that traditional Chinese medicine has a curative effect on Hp and repeated infections.^[1,12,23–25]^ Traditional Chinese medicine has a superior curative effect in treating gastropathy, paying attention to protecting the function of the spleen and stomach, and has few adverse reactions. In the history of the development of Chinese medicine, there is no saying that Hp infection. According to the clinical symptoms of patients with Hp infection, Chinese medicine classified it as “stomach pain”, “acid reflux”, “vomiting”, “hiccup”, “noisy”, and “fullness”. As a pathogenic microorganism, Hp can be classified into the category of “damp-heat pathogens” in traditional Chinese medicine, which has the property of “toxicity”.^[23]^ On the basis of 4 diagnosis and syndrome differentiation, Chinese medicine believes that Hp infection is related to living habits such as spicy and greasy diet, which is easily affected by healthy qi deficiency and pathogenic factors. The pathogenesis includes weakness of the spleen and stomach, damp-heat block, stagnation of qi and blood stasis, stagnation of liver qi, etc.^[26–29]^ Most of the treatment methods are to eliminate pathogenic factors, improve stomach microenvironment, protect the righteousness and create an environment unsuitable for Hp. Traditional Chinese medicine treatment is mainly focuses on clearing away heat and toxic materials, aromatic eliminating dampness, strengthening spleen and benefiting qi. There are also clinical studies that single Chinese medicines have antibacterial effects, such as Coptis, Cortex Phellodendri, and Dandelion have direct antibacterial effects.^[23,30–32]^ Chinese medicine has obvious advantages, but it has not been developed and utilized to a greater extent. The main purpose of this study is to make a comprehensive and systematic evaluation and meta-analysis of the treatment of Hp infection by traditional Chinese medicine, so as to determine the curative effect of traditional Chinese medicine on Hp infection and provide clinical evidence.

## 2 Methods and analysis

This protocol is conducted according to the Preferred Reporting Items for Systematic Reviews and Meta-Analysis Protocol (PRISMA-P) statement guidelines and the Cochrane Handbook for Systematic Reviews of Interventions. This systematic review protocol has been registered on INPLASY as INPLASY2020120057 (doi: 10.37766/inplasy2020.12.0057)

### 2.1 Inclusion and exclusion criteria

#### 2.1.1 Types of studies

This study only includes human randomized controlled trials, and there are no restrictions on language, dissemination date or publication type. Non-randomized controlled trials, quasi-randomized controlled trials, retrospective studies, retrospective studies, case reports, non-controlled trials and animal mechanism studies will be excluded from the systematic review. For inclusion in the trials, researchers need to accurately report randomized methods, diagnostic criteria, intervention details and efficacy evaluation. The duration of treatment and follow-up is infinite.

#### 2.1.2 Types of participants

All patients who have been diagnosed with Hp will be included. There are no restrictions on age, sex, region, race, belief, race, origin and disease course.

#### 2.1.3 Types of interventions

##### 2.1.3.1 Experimental intervention

The experimental group mainly treated Hp infection with traditional Chinese medicine. The use of traditional Chinese medicine is limited to prescription medicines and proprietary Chinese medicines. Prescription drugs need a definite dose, but there are no restrictions on the ingredients, dosage form and doses. For the dosage, such as decoction, granule, pill, powder, etc, other types of traditional Chinese medicine treatment, such as traditional Chinese medicine injections, acupuncture, massage, cupping, etc, will not be included.

##### 2.1.3.2 Comparison interventions

The control group was given conventional western medicines combined with antibiotics, proton pump inhibitors and aluminate preparations. There are no restrictions on specific drugs, doses and methods. If the control group is treated with Chinese medicine, the study will be excluded.

#### 2.1.4 Types of outcome measures

The DOI value of C^13^ and C^14^ breath test decreased or was negative, the antibody of Hp in serum, bacterial culture in gastric mucosa and enzyme test in uremia were negative.

### 2.2 Data sources and search strategy

Relevant research will be conducted on the following databases from establishment to December 2020: Science Net, PubMed, Science Direct, Wanfang Data Knowledge Service Platform, China Biomedical Literature Database (CBM), China Science Journal Database (VIP Database), China National Knowledge Infrastructure (CNKI) and EMBASE. Two reviewers (ZM and YJ) will independently search the research. Any discrepancies will be resolved through discussion with the first author (ZM). We will also search the ongoing trial registrations of the National Institutes of Health, the WHO International Clinical Trials Registration Platform, the Chinese Clinical Trial Registry and Google Scholar to find any relevant ongoing or unpublished trials. For a comprehensive search, we will adopt a search strategy that combines MeSH words and free words. Table 1 shows PubMed's search strategy and the modified search strategy will be applied to other databases.

**Table 1:** Search strategy used in PubMed database.

Number	Search terms
1	Traditional Chinese Medicine(MeSH)
2	(Zhong yi xue OR Chinese herbal medicine)
3	(Single medicine OR Compound prescription):ti.ab
4	1 OR 2 OR 3
5	Helicobacter pylori MeSH)
6	(Hp OR Helicobacter pylori infection OR Eradication of Helicobacter pylori):ti.ab
7	5 OR 6
8	(Randomized controlled trial): pt
9	(Randomized OR placebo):ti.ab
10	8 OR 9
11	4 and 7 and 10

### 2.3 Data collection and analysis

#### 2.3.1 Study selection

**Figure 1:**
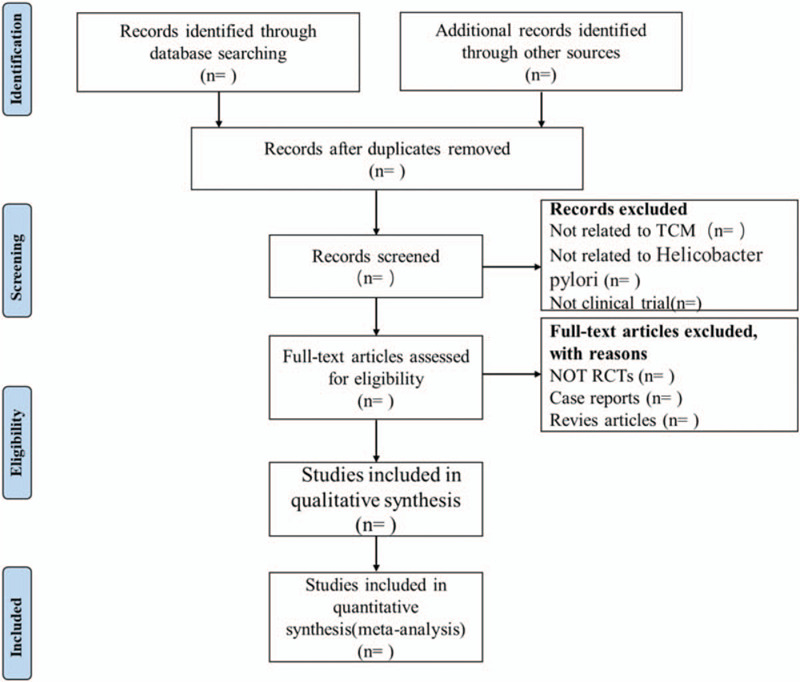
Flow diagram of study selection process.

Two researchers will independently evaluate all relevant studies and review the titles and abstracts to select qualified articles that meet the inclusion criteria. The full text of the article will be reviewed for further evaluation. If there are differences between the review authors, an agreement will be reached through discussion with the corresponding author (YJ). The procedure selected for the study will be summarized through the use of system reviews and preferred reporting items in the Meta-Analytics protocol flow chart (Fig. 1).

#### 2.3.2 Data extraction and management

Before data extraction, a standard data extraction table (Excel) containing the specified results will be created based on the included content. Then, 2 reviewers (ZF and ZC) will independently extract data such as first author, publication year, diagnosis information, disease course, sample size, age, intervention measures, control and results, treatment time, follow-up time, and adverse events. If necessary, any differences will be resolved through discussion or negotiation between the 2 reviewers. The final decision of the third reviewer (YJ) will be sought. When the data of the article is insufficient or ambiguous, one of the authors will contact the original author to request detailed information about the research by email or telephone or estimated data.

#### 2.3.3 Risk of bias assessment

In order to assess the methodological quality of the included studies, 2 reviewers (ZM and ZF) will independently use the Cochrane risk of bias tool to examine 7 aspects: random sequence generation, allocation hiding, blindness of participants and personnel, blindness of result evaluation, incomplete data Evaluations, selective results reports and other sources of bias.

#### 2.3.4 Measures of therapeutic effect

If the studies can be combined, we will conduct a meta-analysis. For dichotomous data, a risk ratio (RR) with a 95% confidence interval (CI) will be calculated. For continuous data, a standardized mean (SMD) with 95% CI will be calculated. If meta-analysis is not possible, we will provide a narrative synthesis of the findings and results.

#### 2.3.5 Unit of analysis issue

Only the date of the first experimental period is considered in the randomized crossover trial. For studies with multiple intervention groups, this review will combine the experimental intervention group and the control intervention group into a single group to avoid analysis problems.

#### 2.3.6 Assessment of heterogeneity

According to the guidelines of the Cochrane Intervention System Evaluation Manual, we will select the *I*^2^ statistic and the chi-square test with a significance level of *P* < .1 to measure the heterogeneity of the study in each analysis. When the *I*^2^ value is less than 50%, the study will not be considered as statistically heterogeneous. When the *I*^2^ value exceeds 50%, there is significant statistical heterogeneity between trials, so meta-analysis will not be performed. And perform subgroup analysis to determine the cause

#### 2.3.7 Assessment of reporting bias

If our review includes enough meta-analyses to be included in the analysis, funnel plots and statistical tests will be generated to analyze potential reporting bias and smaller research effects.

#### 2.3.8 Data synthesis and analysis

When *I*^2^ < 50% is considered to have no evidence of significant statistical heterogeneity, the fixed effects model will be used to merge the data. When *I*^2^ ≥ 50% is regarded as substantial statistical heterogeneity, random effects models will be used to synthesize the data and draw conclusions more cautiously. If the data is not suitable for combining quantitative synthesis, in this case, a narrative description of the system and the information provided in this article will be provided to summarize and explain the characteristics and findings of each study.

#### 2.3.9 Subgroup analysis

To identify substantial heterogeneity, subgroup analysis will be implemented according to characteristics of patients, type of intervention and outcome measures.

#### 2.3.10 Sensitivity analysis

When studies are adequate, sensitivity analysis will be adopted for primary outcomes to explore the robustness of conclusions if feasible, and assess the impact of methodological quality, sample size and missing data. Sensitivity analysis will be conducted by removing lower quality studies if heterogeneity remains after subgroup analysis or studies with incomplete results according to the STRICTA checklist. The meta-analysis will be carried out again after trials of lower quality have been excluded. The results of these meta-analyses then will be compared and discussed according to their sample size, the strength of evidence and influence on the pooled effect size. However, if all included studies have a high risk of bias, we will not carry out sensitivity analyses.

#### 2.3.11 Grading the quality of evidence

The GRADE system will be used for evaluating the quality of evidence in systematic reviews. The evaluation included bias risk; heterogeneity; indirectness; imprecision; publication bias. And each level of evidence will be made “very low,” “low,” “moderate,” or “high” judgment.

## 3 Discussion

Helicobacter pylori (Hp) is a slightly aerobic Gram-negative bacteria, which adheres to the gastric mucosa and damages it, resulting in chronic gastritis, gastroduodenal ulcer, intestinal metaplasia and gastric cancer. The conventional treatment methods for Hp infection include antibiotics, proton pump inhibitors and aluminum bismuth preparations, but they are resistant to drug and re-positive. In recent years, traditional Chinese medicine has been widely used in Hp infection. The purpose of this systematic analysis is to evaluate the efficacy of traditional Chinese medicine in eradicating Hp infection, explore the application of traditional Chinese medicine in improving gastric mucosal environment combined with traditional Chinese medicine antibacterial drugs in patients with Hp infection, and screen out traditional Chinese medicines with antibacterial effects. Research and development of new drugs. The results of the report will be circulated after peer review and publication.

## Author contributions

**Conceptualization:** Mao Zhao, Yong Jiang.

**Data curation:** Zhaoxing Chen, Zhipeng Fan.

**Formal analysis:** Mao Zhao, Yuchang Jiang, Yong Jiang.

**Funding acquisition:** Yong Jiang.

**Investigation:** Mao Zhao, Yuchang Jiang.

**Methodology:** Mao Zhao, Yong Jiang.

**Software:** Zhipeng Fan.

**Supervision:** Yong Jiang.

**Writing – original draft:** Mao Zhao.

**Writing – review & editing:** Zhipeng Fan, Yong Jiang.

## References

[1] Chu ZR Research progress of TCM treatment of Helicobacter pylori infection-related diseases. Chin J Clin Res 2019;12:321731–4.

[2] Bao MJ Research progress on prevention and treatment of Helicobacter pylori. Food Drug 2017;19:171–5.

[3] Savoldi A Carrara E Graham DY Prevalence of antibiotic resistance in Helicobacter pylori: a systematic review and meta-analysis in World Health Organization regions. Gastroenterology 2018;155:1372–82. 29990487 10.1053/j.gastro.2018.07.007 PMC6905086

[4] Zhou R Cao F Liu Y Experimental study on the bacteriostasis of Fuling Gancao Decoction and single Chinese medicine on Helicobacter pylori in vitro. Asia Pac Tradit Med 2020;16:217–20.

[5] Hooi JKY Lai WY Ng WK Global Prevalence of Helicobacter pylori Infection: Systematic Review and Meta-analysis. Gastroenterology 2017;doi: 10.1053/j.gastro.2017.04.022. 10.1053/j.gastro.2017.04.022 28456631

[6] Yi-Chia Lee M Chiang T Chou C Association between Helicobacter pylori eradication and gastric cancer incidence. Gastroenterology 2016;150:1113–24.e5. 26836587 10.1053/j.gastro.2016.01.028

[7] Xu FF Zhou Z Current research status of traditional Chinese medicine against Helicobacter pylori infection. J Pract Tradit Chin Med 2016;32:9945–7.

[8] Jin D Liu HW Research progress of TCM treatment of Helicobacter pylori. Guang Ming Tradit Chin Med 2016;31:182756–8.

[9] Cai HL Xiao LR Lin D Recent development of traditional Chinese medicine treatment of Helicobacter pylori infection. Strait Pharm J 2014;26:797–1000.

[10] Wang Z Clinical analysis of Huangqi Jianzhong Decoction in treating Helicobacter pylori positive gastric ulcer. Med Res 2019;3:816–7.

[11] Li WS Clinical observation on treatment of 16 cases of chronic gastritis infected by drug-resistant Helicobacter pylori with traditional Chinese medicine. World Latest Med Inform 2019;19:29154–9.

[12] Di W Zhang WJ Analysis on the rule of traditional Chinese medicine for the treatment of Helicobacter pylori infection. Chin Med Herald 2018;15:36121–4.

[13] Chen XH You YH Observation on the effect of self-made Xiangling Decoction on Helicobacter pylori positive gastric ulcer. Chin Med Technol 2018;25:5733–4.

[14] Xue SM Analysis of curative effect of Mieyou Decoction on damp-heat Helicobacter pylori-related gastritis. Guang Ming Tradit Chin Med 2017;32:166–7.

[15] Wang CH Qi HJ Research progress of traditional Chinese medicine against Helicobacter pylori. Shaanxi Tradit Chin Med 2011;32:6763–5.

[16] Zhang QY Clinical Study of Xinkaikujiang Therapy for the Treatment of Cold-Heat Mixed Helicobacter Pylori Infection. Beijing: University of Chinese Medicine; 2017.

[17] Zhang FQ Bai G Treatment of Helicobacter pylori-related gastropathy from “toxin and depression”. Liaoning J Tradit Chin Med 2015;42:71244–5.

[18] Gan YH Yan H Cheng Z Research progress of traditional Chinese medicine in the treatment of Helicobacter pylori infection. Hunan J Tradit Chin Med 2014;30:2141–3.

[19] Yang SX Zhou FX Effect of Jianpi Huayu Qingyou Decoction on HP infectious chronic gastritis. Chin J Chin Med 2011;26:159993–4.

[20] Li Y Liu HY Research progress of traditional Chinese medicine on Helicobacter pylori-related gastritis. Mod J Integr Tradit Chin West Med 2011;27:203500–2.

[21] Pan H Research progress on treatment of Helicobacter pylori with Chinese and Western medicine. J Pract Tradit Chin Med 2011;25:249–50.

[22] Liu F Zhang BP Xie QP New progress in treatment of Hp-related peptic ulcer with traditional Chinese medicine. J New Chin Med 2011;43:01122–4.

[23] Qiu CY Study on the regularity of Chinese medicine prescriptions in the treatment of Helicobacter pylori infection in recent ten years. Tradit Chin Med Clin Res 2020;12:2917–9.

[24] Cai YY Research Progress of Helicobacter Pylori in Traditional Chinese Medicine. Beijing: Beijing University of Chinese Medicine; 2012.

[25] Guo XD Study on the Eradication of Helicobacter Pylori with Chinese Medicine (Bacteriostatic Experiment). Liaoning: Liaoning University of Traditional Chinese Medicine; 2011.

[26] Wang WD Observation on the clinical effect of supplementing qi, activating blood and resolving phlegm in the treatment of Hp infection. J Chron Dis 2019;20:6868–9.

[27] Lv X To explore the efficacy of the method of replenishing qi and detoxification in the treatment of helicobacter pylori infection of stomach. World Latest Med Inform 2016;16:102154–6.

[28] Ding Y Observation on the effect of traditional Chinese medicine for invigorating the spleen and dissipating dampness in intervention of recurrent Helicobacter pylori infection. Shanghai Pharm 2014;35:2026–7.

[29] Li XM Clinical Study on Qingwei Qushi Decoction in Treating Hp-related Chronic Gastritis of Liver Stagnation and Spleen Deficiency with Damp-heat. Guangzhou: Guangzhou University of Chinese Medicine; 2011.

[30] Zhang L Research progress of traditional Chinese medicine against Helicobacter pylori. Res Integr Tradit Chin West Med 2015;7:2106–8.

[31] Wang BL Chen HG Treatment of Helicobacter pylori-related infections with traditional Chinese medicine. Clin J Chin Med 2015;7:1626–8.

[32] Ping LH Observation on the effect of self-made Huanglian Decoction on Helicobacter pylori infection complicated with gastric ulcer. Yunnan J Tradit Chin Med 2014;35:226–7.

